# The Interaction of Age and Type 2 Diabetes on Executive Function and Memory in Persons Aged 35 Years or Older

**DOI:** 10.1371/journal.pone.0082991

**Published:** 2013-12-18

**Authors:** Marlise E. A. van Eersel, Hanneke Joosten, Ron T. Gansevoort, Robin P. F. Dullaart, Joris P. J. Slaets, Gerbrand J. Izaks

**Affiliations:** 1 University of Groningen, University Medical Center Groningen, University Center for Geriatric Medicine, Groningen, The Netherlands; 2 University of Groningen, University Medical Center Groningen, Department of Nephrology, Groningen, The Netherlands; 3 University of Groningen, University Medical Center Groningen, Department of Endocrinology, Groningen, The Netherlands; 4 University of Groningen, University Medical Center Groningen, Alzheimer Center Groningen, Groningen, The Netherlands; CUNY, United States of Ameirca

## Abstract

It is generally assumed that type 2 diabetes increases the risk of cognitive dysfunction in old age. As type 2 diabetes is frequently diagnosed before the age of 50, diabetes-related cognitive dysfunction may also occur before the age of 50. Therefore, we investigated the association of type 2 diabetes with cognitive function in people aged 35–82 years. In a cross-sectional study comprising 4,135 participants of the Prevention of Renal and Vascular ENd-stage Disease study (52% men; mean age (SD), 55 (12) years) diabetes was defined according to the criteria of the American Diabetes Association. Executive function was measured with the Ruff Figural Fluency Test (RFFT; worst score, 0 points; best score, 175 points), and memory was measured with the Visual Association Test (VAT; worst score, 0 points; best score, 12 points). The association of diabetes with cognitive function was investigated with multiple linear or, if appropriate, logistic regression analysis adjusting for other cardiovascular risk factors and *APOE* ε4 carriership. Type 2 diabetes was ascertained in 264 individuals (6%). Persons with diabetes had lower RFFT scores than persons without diabetes: mean (SD), 51 (19) vs. 70 (26) points (*p*<0.001). The difference in RFFT score was largest at age 35–44 years (mean difference 32 points; 95% CI, 15 to 49; *p*<0.001) and gradually decreased with increasing age. The association of diabetes with RFFT score was not modified by *APOE* ε4 carriership. Similar results were found for VAT score as outcome measure although these results were only borderline statistically significant (*p*≤0.10). In conclusion, type 2 diabetes was associated with cognitive dysfunction, especially in young adults. This was independent of other cardiovascular risk factors and *APOE* ε4 carriership.

## Introduction

The global prevalence of diabetes is expected to rise over the next twenty years due to population growth, the growth of life expectancy and the increasing prevalence of obesity and physical inactivity. It is estimated that the total number of people with diabetes will increase from 171 million in 2000 to 366 million in 2030 [1]. Diabetes causes microvascular and macrovascular damage resulting in nephropathy, retinopathy and cardiovascular disease [2], [3]. In addition, chronic hyperglycemia can lead to microvascular changes in the brain [3], probably leading to brain atrophy and white matter lesions. In autopsy and imaging studies, diabetes is associated with cerebral atrophy and cerebrovascular lesions [4], [5]. Therefore, diabetes may be an important causal factor of cognitive decline and development of dementia in late life.

Several studies have investigated the association of diabetes with cognitive decline. It was not only shown that diabetes increases the risk of dementia [6], [7] but also accelerates cognitive decline in older persons without dementia [8]. As diabetes is frequently diagnosed before the age of 50 years, it is likely that the accelerated cognitive decline in diabetes already occurs at a relatively young age [9]. A better understanding of the association of diabetes with cognitive decline may contribute to early prevention of severe cognitive dysfunction later in life. However, five large cross-sectional and longitudinal population-based studies in middle-aged people showed divergent results [10]–[14]. This might be due to several factors such as the relatively low prevalence of diabetes in young people [11], the drop-out of subjects with diabetes during longitudinal follow-up [12]–[14], or the different degree of adjustment for other cardiovascular risk factors [10]–[14]. Moreover, it was recently suggested that the effect of diabetes on cognitive function is possibly modified by *APOE* ε4 carriership in middle-aged and old people [15], [16]. Up till now, however, it is not clear if *APOE* ε4 carriership has a similar effect in younger persons with diabetes.

The aim of this study was to investigate the association of type 2 diabetes with cognitive function in a large community-based sample of people aged 35 years or older. The total sample included 4,135 participants of whom 264 persons had diabetes. In all persons, we established cognitive performance on executive function and memory tests, cardiovascular risk factors and *APOE* ε4 carriership.

## Methods

### Study population

The study population included all participants of the third survey of the Prevention of REnal and Vascular ENd-stage Disease (PREVEND) cohort (N_total_  =  5,862). The PREVEND study was designed to investigate prospectively the natural course of microalbuminuria and its association with renal and cardiovascular diseases in the general population. In brief, during 1997–1998, all 85,421 inhabitants of the city of Groningen, the Netherlands, aged 28–75 years were invited to participate in this study. A total of 40,856 (48%) people responded. Participants were selected based on their urinary albumin excretion (UAC): 3,395 with UAE <10 mg/dl and 7,768 with UAE >10 mg/dl. People with insulin-dependent diabetes were excluded. A total of 8,592 participants completed the baseline survey and were followed over time. During follow-up, 6,984 participants completed the second survey in 2001–2003, and 5,862 the third survey in 2003–2006 (80% and 68% of the cohort at baseline, respectively). All surveys included assessments of demographic, anthropometric and cardiovascular risk factors, and measurements of hematological and biochemical parameters. Cognitive function tests for executive function and memory were introduced at the third survey of the PREVEND study. Further details of the PREVEND study can be found in Mahmoodi et al. and Lambers Heersink et al. [17], [18].

### Ethics Statement

The PREVEND study was approved by the medical ethics committee of the University Medical Center Groningen, The Netherlands, and conducted in accordance with the guidelines of the Helsinki declaration. All participants gave written informed consent.

### Executive Function

Executive function was measured with the Ruff Figural Fluency Test (RFFT) [19], [20]. The RFFT requires the participants to draw as many designs as possible within a set time limit while avoiding repetitions of designs [19], [20]. The RFFT is generally seen as a measure of executive function but provides information regarding different cognitive abilities such as planning strategies, divergent thinking and the ability to shift between different cognitive tasks [19], [20]. The RFFT is sensitive to changes in cognitive function in young and old persons [20], [21]. The main outcome measure of the RFFT is the total number of unique designs, which varies from 0 points (worst score) to 175 points (best score).

### Memory

table-3-captionMemory was measured with the Visual Association Test (VAT) [22]. The VAT is a brief learning task that is designed to detect (impaired) memory including anterograde amnesia. The test consists of six drawnings of two interacting objects. The person is asked to name each object and, later, as a single object of the two is presented the person is asked to name the other object. The lowest (worst) score is 0 points, the highest (best) score is 12 points [22].

### Type 2 Diabetes

table-3-captionType 2 diabetes was defined as a fasting glucose ≥7.0 mmol/L (126 mg/dl) or a non-fasting glucose ≥11.1 mmol/L (200 mg/dl), the use of glucose-lowering medication or self-reported diabetes in a questionnaire at the third survey [23]. Plasma glucose was measured by dry chemistry (Eastman Kodak, Rochester, NY). Data on actual use of glucose-lowering medication were obtained from the InterAction DataBase (www.iadb.nl) that comprised pharmacy-dispending data from regional community pharmacies [24].

### APOE genotype

Genotyping of *APOE* allele status (rs429358 and rs7412) was performed using polymerase chain reaction (PCR) after DNA extraction from a whole blood sample [25]. Participants were categorized as *APOE* ε4 carriers (allele combinations ε2/ε4 or ε3/ε4 or ε4/ε4) or noncarriers (allele ε2/ε2 or ε2/ε3 or ε3/ε3) [25].

### Other measurements

Several demographic and cardiovascular risk factors are associated with cognitive decline and with diabetes. Data on age, gender and educational level were obtained from a questionnaire at baseline. Educational level was divided into four groups according to the International Standard Classification of Education (ISCED): primary school (0 to 8 years of education; ISCED 0-1), lower secondary education (9 to 12 years of education; ISCED 2), higher secondary education (13 to 15 years of education; ISCED 3-4), and university (≥16 years of education; ISCED 5) [26]. A history of cardiovascular disease was defined as a prior cardiac, cerebrovascular or peripheral vascular event requiring hospitalization (at baseline, data were obtained from a questionnaire and during follow-up until the third survey from the Dutch national registry of hospital discharge diagnoses). At the third survey all the cardiovascular risk factors were measured. Smoking was defined as current smoker based on self-report. Blood pressure was automatically measured (Dinamap) in a supine position during ten minutes and reported as the average of the two last measurements. Fasting blood was drawn for the determination of total cholesterol, HDL cholesterol and glucose. Non-HDL cholesterol was calculated as total cholesterol minus HDL cholesterol. Microalbuminuria was determined by nephelometry in two consecutive 24-hour urine samples, and defined as albuminuria ≥30 mg/24 hours [18].

### Statistical analysis

Parametric data are presented as mean and standard deviation (SD) and nonparametric data as median and interquartile range (IQR). Differences between groups were tested by the independent-samples t-test for parametric data and by Mann-Whitney *U* test for nonparametric data. Differences in proportion were tested by Chi-Square test. Trends in proportion across groups were tested by Linear-by-Linear Association test.

The association of RFFT score with type 2 diabetes was analyzed by multiple linear regression analysis. RFFT score (total number of unique designs) was the dependent variable, type 2 diabetes (yes/no) was the independent variable. The full regression model also included the independent variables age, gender, educational level, BMI, smoking, systolic blood pressure, HDL cholesterol, non-HDL cholesterol, microalbuminuria and *APOE* ε4 carriership to adjust for possible confounders. Interaction between diabetes and age was tested by entering the product term type 2 diabetes x age into the regression model. Similarly, interaction between diabetes and *APOE* ε4 carriership was tested by entering type 2 diabetes x *APOE* ε4 carriership into the model. In all regression models, the variables RFFT score, age, BMI, systolic blood pressure, HDL cholesterol and non-HDL cholesterol were entered as continuous variables. All other variables were entered as categorical variables. The level of statistical significance was set at 0.05.

Similar analyses were performed for VAT score as outcome measure. Because of its skewed distribution VAT score was dichotomized at the median into low performance (≤10 points) and high performance (≥11 points). The association of VAT performance with type 2 diabetes was analyzed by logistic regression analysis. All analyses were performed using IBM SPSS Statistics 20.0 (IBM, Amonk, NY).

### Sensitivity analyses

As a consequence of its design, the participants of the third survey of the PREVEND study had a somewhat higher prevalence of microalbuminuria than the general population (10% vs. 8%, respectively) [18], [27]. Because this may influence data analyses, the multiple linear regression analyses for RFFT score were repeated in a subset of the PREVEND cohort, the Groningen Random Sample (N = 1,651), which had a similar prevalence of microalbuminuria (8%) and other cardiovascular risk factors as the general population [18]. However, the logistic regression analyses for VAT score were not repeated in the Groningen Random Sample because the number of participants with type 2 diabetes and low performance on the VAT was very small in this sample (N = 22). Additionally, the analyses were repeated after exclusion of all *APOE* ε2 carriers (allele combinations ε2/ε2, ε2/ε3 and ε2/ε4) because the *APOE* ε2 allele appears to reduce the risk of Alzheimer’s disease [28], and the effect of *APOE* ε2/4 genotype on cognitive function is unclear (N = 3,185).

## Results

### Study population

Of the 5,862 participants of the third survey, 4,158 participants completed the RFFT (71%). A total of 1,271 participants (22%) refused to perform the RFFT and 433 (7%) had incomplete RFFT data. Of those with a complete RFFT, twenty participants (0.5%) were excluded because their educational level was not known and three participants (0.1%) because their age was younger than 35 years and their number too small to form a separate age group. Thus, the total study population included 4,135 persons. The mean age (SD) was 55 (12) years, 52% was male and 96% was of Western-European descent (Table 1).

**Table 1:** Characteristics of the study population.

	All	Type 2 Diabetes		
		No	Yes	*p* a
**N (%)**	4135 (100)	3871 (100)	264 (100)^b^	N/A
**Gender, N (%)**				<0.001
Men	2157 (52)	1991 (51)	166 (63)	
Women	1978 (48)	1880 (49)	98 (37)	
**Age (years), mean (SD)**	55 (12)	54 (11)	64 (10)	<0.001
**Educational level, N (%)**				<0.001
Primary school	406 (10)	349 (9)	57 (22)	
Lower secondary education	1225 (29)	1120 (29)	105 (40)	
Higher secondary education	1108 (27)	1054 (27)	54 (20)	
University	1396 (34)	1348 (35)	48 (18)	
**Cardiovascular disease history, N (%)**	296 (7)	250 (7)	46 (17)	<0.001
**Cardiovascular risk factors**				
Smoking, N (%)	979 (24)	926 (24)	53 (20)	0.17
Body Mass Index (kg/m^2^), mean (SD)	27 (4)	27 (4)	30 (5)	<0.001
Systolic blood pressure (mmHg), mean (SD)	126 (18)	125 (18)	136 (19)	<0.001
Glucose (mmol/L), mean (SD)	4.88 (0.95)	4.72 (0.58)	7.24 (1.82)	<0.001
Total cholesterol (mmol/L), mean (SD)	5.36 (1.06)	5.41 (1.03)	4.69 (1.22)	<0.001
HDL cholesterol (mmol/L), mean (SD)	1.41 (0.38)	1.42 (0.38)	1.17 (0.33)	<0.001
Non-HDL cholesterol (mmol/L), mean (SD)	3.96 (1.03)	3.99 (1.01)	3.51 (1.20)	<0.001
Microalbuminuria, N (%)	600 (15)	498 (13)	102 (39)	<0.001
***APOE*** ** ε4 carriership** c **, N (%)**	1166 (28)	1089 (28)	77 (29)	0.69
**RFFT score (points), mean (SD)**	69 (26)	70 (26)	51 (19)	<0.001
**VAT score (points)** d **, median (IQR)**	10 (9–11)	10 (9–11)	9 (8–11)	<0.001
Low performance (≤10 points), N (%)	2425 (59)	2232 (58)	193 (73)	<0.001
High performance (≥11 points), N (%)	1628 (39)	1561 (40)	67 (25)	

Abbreviations: RFFT, Ruff Figural Fluency Test; VAT, Visual Association Test; SD, standard deviation; IQR, interquartile range; N/A, not applicable.

^a^ *p*-values refer to comparisons between persons with and without diabetes.

b Prevalence of type 2 diabetes in total study population was 6% (N = 264).

c Including ε2/ε4, ε3/ε4 and ε4/ε4. *APOE* ε4 carriership was unknown for 280 persons of the total study population (7%): with diabetes, 18 (7%) and without diabetes, 262 (7%).

d VAT score was unknown for 82 persons of the total study population (2%): with diabetes, 4 (2%) and without diabetes, 78 (2%).

The total study population included 264 persons with type 2 diabetes (6%). Thirty-seven persons (14%) used insulin, 152 persons (58%) used oral glucose-lowering medication, and 4 (2%) used a combination of the two. Persons with diabetes were older and had a lower educational level compared to people without diabetes (Table 1). Also, persons with diabetes had a higher prevalence of cardiovascular disease history, hypertension and microalbuminuria and worse values for most cardiovascular risk factors. Two persons with diabetes (0.8%) had a glucose value below 4.00 mmol/L but no symptoms of hypoglycemia.

Participants, who did not perform the RFFT were slightly older (mean age [SD], 56 [12] vs. 55 [12] years; *p*<0.001), were more often women (52% vs. 48%; *p*<0.001) and had a lower educational level (*p*<0.001). There were no statistically significant differences in the prevalence of diabetes (*p* = 0.48*)*, other cardiovascular risk factors (*p*≥0.39) or *APOE* ε4 carriership (*p* = 0.11).

### RFFT and Type 2 Diabetes

**Figure 1:**
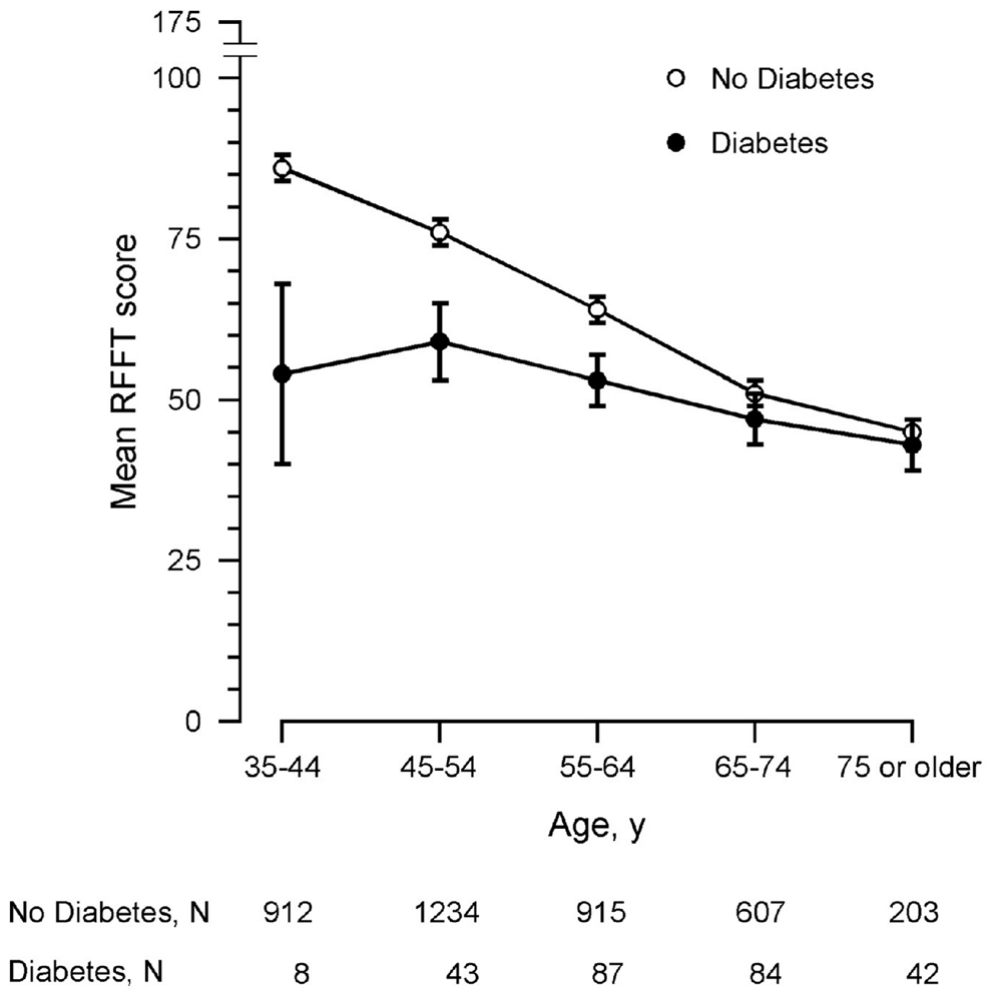
RFFT score dependent on age and the presence of type 2 diabetes. For clarity, data are presented as mean and 95% confidence interval in ten year age groups (data unadjusted for possible confounders). Abbreviation: RFFT, Ruff Figural Fluency Test.

Persons with diabetes had lower RFFT scores than persons without diabetes: mean (SD), 51 (19) points vs. 70 (26) points, respectively (*p*<0.001). However, the difference in RFFT score between persons with and without diabetes was clearly dependent on age, since it diminished from 32 points (95% CI, 15 to 49; *p*<0.001) in persons aged 35–44 years to 2 points (95% CI, –4 to 8; *p* = 0.60) in persons aged 75 years and older (Figure 1). This was confirmed by multiple linear regression analysis that did not only show a statistically significant effect for type 2 diabetes (B-coefficient [95% CI],–40.27 [–57.20 to –23.35]; *p*<0.001), but also for the interaction type 2 diabetes x age (Table 2). The difference in RFFT scores between persons with and without diabetes decreased by 0.48 point per one-year increment of age (Table 2). The negative association of diabetes with RFFT score and the interaction between diabetes and age were also found after adjustment for several cardiovascular risk factors. There was no statistically significant interaction between type 2 diabetes and *APOE* ε4 carriership: B-coefficient for diabetes –38.03 (95% CI, –55.93 to –20.12; *p*<0.001), for *APOE* ε4 carriership, 0.22 (95% CI, –1.27 to 1.71; *p* = 0.77), and for type 2 diabetes x *APOE* ε4 carriership, 4.12 (95% CI, –1.69 to 9.92; *p* = 0.17).

**Table 2:** Multiple linear regression analysis of Ruff Figural Fluency Test score on type 2 diabetes and age.

	Model 1^a^			Model 2^a^			Model 3^a^		
	B	95% CI	*p*	B	95% CI	*p*	B	95% CI	*p*
Age (years)	–0.90	–0.96 to –0.84	<0.001	–0.92	–0.98 to –0.86	<0.001	–0.92	–0.99 to –0.85	<0.001
Gender (women vs. men)	0.09	–1.18 to 1.36	0.89	0.01	–1.25 to 1.28	0.98	–0.78	–2.23 to 0.66	0.29
Educational level (vs. primary school)									
Lower secondary education	5.88	3.55 to 8.22	<0.001	6.01	3.68 to 8.34	<0.001	5.37	3.00 to 7.74	<0.001
Higher secondary education	13.68	11.25 to 16.11	<0.001	13.67	11.25 to 16.10	<0.001	12.80	10.32 to 15.27	<0.001
University	24.19	21.80 to 26.58	<0.001	24.16	21.77 to 26.54	<0.001	22.55	20.07 to 25.02	<0.001
Type 2 diabetes (yes vs. no)	–6.27	–8.90 to –3.63	<0.001	–40.27	–57.20 to –23.35	<0.001	–35.91	–53.01 to –18.80	<0.001
Type 2 diabetes x age	-	-	-	0.54	0.27 to 0.80	<0.001	0.48	0.22 to 0.75	<0.001
BMI (kg/m^2^)	-	-	-	-	-	-	–0.15	–0.31 to 0.01	0.07
Smoker (yes vs. no)	-	-	-	-	-	-	–2.88	–4.42 to –1.34	<0.001
Systolic BP (mmHg)	-	-	-	-	-	-	–0.01	–0.06 to 0.03	0.52
HDL cholesterol (mmol/L)	-	-	-	-	-	-	2.31	0.40 to 4.22	0.02
Non-HDL cholesterol (mmol/L)	-	-	-	-	-	-	0.11	–0.53 to 0.74	0.74
Microalbuminuria (yes vs. no)	-	-	-	-	-	-	–1.39	–3.32 to 0.53	0.16

Abbreviations: B, unstandardized B-coefficient; CI, confidence interval; Systolic BP, Systolic Blood Pressure.

a For all models: adjusted R^2^, 0.37; residual standard deviation, 21.

### VAT and Type 2 Diabetes

**Figure 2:**
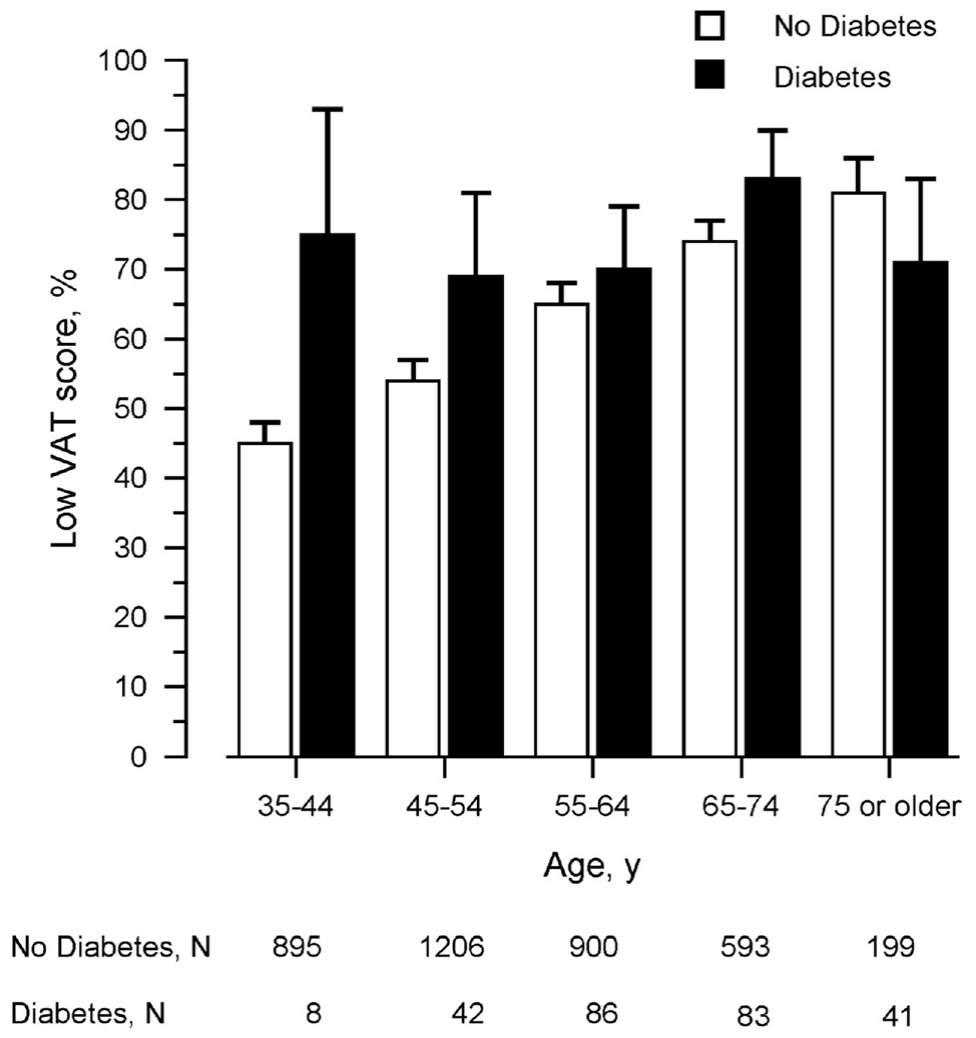
Percentage of persons with low performance on the VAT dependent on age and the presence of type 2 diabetes. Bars represent 95% confidence intervals. Data unadjusted for possible confounders. Abbreviation: VAT, Visual Association Test.

Similar results were found for the association of VAT score with type 2 diabetes. VAT scores were obtained in 4,053 persons of whom 2,425 (59%) had a low performance (Table 1). Overall, the percentage of persons with low performance was higher in persons with diabetes than in persons without diabetes: 73% vs. 58% (*p*<0.001). In persons without diabetes, the percentage of persons with low performance gradually increased from 45% in age group 35–44 years to 81% in age group 75 years or older (*p*_trend_<0.001) (Figure 2). However, in persons with type 2 diabetes, the percentage of persons with low performance was similar in all age groups and varied between 69–83% (*p*_trend_ = 0.37). Thus, the difference between persons with and without diabetes in performance on the VAT was largest in the youngest age group. The association of type 2 diabetes with low performance on the VAT as well as the interaction of type 2 diabetes x age was also found in logistic regression analysis after adjustment for several cardiovascular risk factors (Table 3). However, the odds ratios were only borderline statistically significant (*p*≤0.10). There was no statistically significant effect of *APOE* ε4 carriership or the interaction of type 2 diabetes x *APOE* ε4 carriership (data not shown).

**Table 3:** Logistic regression analysis of low Visual Association Test performance on type 2 diabetes and age.

	Model 1^a^			Model 2^a^			Model 3^a^		
	OR	95% CI	*p*	OR	95% CI	*p*	OR	95% CI	*p*
Age (years)	1.04	1.03 to 1.05	<0.001	1.04	1.03 to 1.05	<0.001	1.04	1.03 to 1.05	<0.001
Gender (women vs. men)	0.65	0.57 to 0.74	<0.001	0.65	0.57 to 0.75	<0.001	0.70	0.61 to 0.82	<0.001
Educational level (vs. primary school)									
Lower secondary education	0.74	0.57 to 0.97	0.03	0.74	0.56 to 0.96	0.02	0.73	0.56 to 0.96	0.03
Higher secondary education	0.66	0.50 to 0.86	0.002	0.66	0.50 to 0.86	0.002	0.66	0.50 to 0.88	0.004
University	0.47	0.36 to 0.61	<0.001	0.47	0.36 to 0.61	<0.001	0.48	0.37 to 0.64	<0.001
Type 2 diabetes (yes vs. no)	1.19	0.88 to 1.61	0.25	6.30	0.95 to 42.02	0.06	5.85	0.86 to 39.88	0.07
Type 2 diabetes x age	-	-	-	0.97	0.95 to 1.00	0.08	0.98	0.95 to 1.01	0.10
BMI (kg/m^2^)	-	-	-	-	-	-	1.00	0.98 to 1.02	0.80
Smoker (yes vs. no)	-	-	-	-	-	-	1.01	0.86 to 1.18	0.95
Systolic BP (mmHg)	-	-	-	-	-	-	1.00	1.00 to 1.00	0.93
HDL cholesterol (mmol/L)	-	-	-	-	-	-	0.79	0.65 to 0.97	0.02
Non-HDL cholesterol (mmol/L)	-	-	-	-	-	-	1.06	0.99 to 1.13	0.12
Microalbuminuria (yes vs. no)	-	-	-	-	-	-	0.90	0.74 to 1.11	0.34

Abbreviations: OR, odds ratio; CI, confidence interval; Systolic BP, Systolic Blood Pressure.

a Nagelkerke R Square for all models, 0.109.

### Sensitivity analyses

Essentially similar results were found if the analyses of the association of RFFT score with type 2 diabetes were repeated in the Groningen Random Sample. In the full regression model, the B-coefficient for type 2 diabetes was –37.90 (95% CI, –69.76 to –6.05; *p* = 0.02), and for type 2 diabetes x age, 0.50 (95% CI, 0.01 to 0.99; *p* = 0.04). If the analyses were repeated after exclusion of all *APOE* ε2 carries, the negative association of diabetes with RFFT score and the interaction between diabetes and age were also found. Comparable to the analyses in the full study population, there was no interaction between type 2 diabetes and *APOE* ε4 carriership (*p* = 0.08). This was also found after exclusion of all *APOE* ε2 carriers (data not shown).

## Discussion

In this large community-based sample, type 2 diabetes was negatively associated with cognitive function, especially executive function and possibly also memory. This negative association was especially found in young people as the difference in cognitive function between persons with and without diabetes decreased with increasing age. In addition, the association of type 2 diabetes with cognitive dysfunction was not modified by *APOE* ε4 carriership.

Most importantly, we found that the difference in cognitive function between persons with and without type 2 diabetes was largest in young persons and decreased with advancing age. This is similar to the finding that the difference in life expectancy between people with and without diabetes is largest in young persons [29]. There are several explanations for this result. First, the onset of type 2 diabetes may be nine to twelve years prior to the clinical diagnosis [30]. It is likely that in this preclinical period, untreated chronic hyperglycemia already causes important and irreversible microvascular changes in the brain [3], [30]. Second, the number of cardiovascular risk factors significantly increases with age in both people with and without diabetes [31]. As cardiovascular risk factors act via shared biological pathways, this probably reduces the difference in cardiovascular risk between the two groups. Finally, both people with and without diabetes undergo age-associated neurodegenerative changes that are caused by other mechanisms such as, for example, oxidative stress, mitochondrial dysfunction and protein misfolding [32]. Therefore, it can be hypothesized that the lack of difference in cognitive function between persons with and without type 2 diabetes in older age groups is attributable to the accumulation of different cerebrovascular and neurodegenerative changes with aging. This hypothesis is further supported by the finding that conventional vascular risk factors may have a different predictive value in young and old persons. Several studies have found, for example, that an increase in cholesterol or Framingham risk score predicts a higher risk of stroke in young persons but not in very old persons [33], [34]. Thus, the effect of risk factors may change over the life course. Accordingly, the effect of type 2 diabetes on brain structure and function may be different in young and old persons.

The results in this study were different from the results in the Third National Health and Nutrition Examination Survey (NHANES III) [10]. In NHANES III, type 2 diabetes was not associated with cognitive function. However, NHANES III included a large proportion of black and Hispanic participants [10]. Compared with white people who have the same cardiovascular risk factors, black and Hispanic people have less carotid and coronary artery calcification [35], [36]. So maybe, the association between diabetes and microvascular changes in the brain, and as a consequence cognitive function, might also be less clear in young and middle-aged black and Hispanic people. Our findings were supported by the Maastricht Aging Study (MAAS) and the Atherosclerosis Risk in Communities (ARIC) cohort study although the adjustment for other cardiovascular risk factors was limited in these studies [11]–[13]. Clearly, adjustment for other cardiovascular risk factors is important as these risk factors are also independently associated with cognitive decline and share common biological pathways with diabetes [37]–[39]. This reasoning was also followed in the longitudinal Doetinchem Cohort Study (DCS) that also found a statistically significant negative association of type 2 diabetes with global cognitive function in young and middle-aged people after adjustment for a large number of well-defined cardiovascular risk factors [14]. Therefore, it is highly likely that type 2 diabetes is associated with cognitive dysfunction in young and middle-aged people.

Several biological mechanisms may explain the relationship between type 2 diabetes and cognitive dysfunction. First, diabetes may accelerate cognitive decline through cerebrovascular disease. It has been found that type 2 diabetes is associated with more cerebral infarcts and white matter lesions [5]. As well, diabetes increases the risk of ischemic stroke two- to fourfold [40]. Second, hyperinsulinemia due to insulin resistance may modulate amyloid-β release and degradation [41]. Additionally, insulin inhibits phophorylation of tau, which leads to an increase in neurofibrillary tangles [41]. Both amyloid-β neuritic plaques and neurofibrillary tangles are the major histopathological features of Alzheimer’s disease [42]. Third, chronic hyperglycemia affects brain tissue through direct toxic effect on neurons by oxidative stress and accumulation of advanced glycation end-products (AGEs) [43]. In addition, chronic hyperglycemia causes microvascular changes as thickening of capillary basement membrane and endothelial cell degeneration resulting in angiopathy and reduced cerebral blood flow [3], [43]. It is plausible to assume that these neurodegenerative and vascular changes are important determinants in the association of type 2 diabetes with cognitive dysfunction.

Recently, it was found that the effect of diabetes on cognitive function may be modified by *APOE* ε4 carriership [16]. However, this was not confirmed in our study. To some extent, this was an unexpected result because both *APOE* ε4 carriership and diabetes are associated with neurodegenerative changes due to amyloid-β and neurofibrillary tangles and vascular changes due atherosclerosis [3], [41], [44]. On the other hand, there also was no difference in cognitive decline between diabetic *APOE* ε4 carriers and noncarriers in the Cardiovascular Health Study (CHS) and in the MAAS study [12], [15]. Therefore, it is still unclear whether *APOE* ε4 carriership modifies the association of diabetes with cognitive dysfunction.

Some limitations of this study have to be noted. First, the lack of a difference in cognitive function between persons with and without diabetes at high age could be due to selection bias as older people with cognitive dysfunction are more likely to refuse study participation than age peers with normal cognitive function [45]. This could have led to some self-selection in our study and consequentially, to an overestimation of cognitive performance in older persons with type 2 diabetes. However, there were no differences in demographic characteristics and cardiovascular risk factors at the third survey between participants with diabetes who did or did not perform the cognitive tests. Also, there are several other studies that only found a weak association between diabetes and cognitive function or dementia [6], [7]. Second, although type 2 diabetes was clearly associated with executive function, the findings on memory were less obvious in our study. Probably, this can be attributed to differences in test characteristics between the two cognitive tests that we used. The executive function test (RFFT) is a sensitive test that does not exhibit a significant floor or ceiling effect [19]–[21]. However, the memory test (VAT) may lack sensitivity to subtle changes in memory in young age groups as it was specifically developed to detect early Alzheimer’s disease [22]. Alzheimer’s disease usually occurs in older age groups. Third, the PREVEND cohort is enriched for persons with microalbuminuria which is associated with cognitive dysfunction [46]. However, the absolute difference in prevalence of microalbuminuria between the PREVEND cohort and the general population was small and a sensitivity analysis in a sample that was representative of the general population showed similar results. Finally, the cross-sectional design may be considered as a limitation because it does not allow determing a causal relationship. Our findings have to be confirmed in a longitudinal study.

The present study also has several strengths. Our study was based on a large community-based sample with a wide age-range and included a large number of young and middle-aged people. Most importantly, the association of cognitive function with diabetes was adjusted for a large number of well-defined and well-measured cardiovascular risk factors as well as *APOE* ε4 carriership.

In conclusion, in this study, type 2 diabetes was negatively associated with cognitive function, especially executive function and possibly also memory. This was independent of cardiovascular risk factors and *APOE* ε4 carriership. The difference in cognitive function between persons with and without type 2 diabetes was largest in the youngest persons, who were aged 35–44 years.

## References

[1] Wild S, Roglic G, Green A, Sicree R, King H (2004) Global prevalence of diabetes: Estimates for the year 2000 and projections for 2030. Diabetes Care 27: 1047–1053. 15111519 10.2337/diacare.27.5.1047

[2] Klein R (1995) Hyperglycemia and microvascular and macrovascular disease in diabetes. Diabetes Care 18: 258–268. 7729308 10.2337/diacare.18.2.258

[3] Beckman JA, Creager MA, Libby P (2002) Diabetes and atherosclerosis: Epidemiology, pathophysiology, and management. JAMA 287: 2570–2581. 12020339 10.1001/jama.287.19.2570

[4] Arvanitakis Z, Schneider JA, Wilson RS, Li Y, Arnold SE, et al (2006) Diabetes is related to cerebral infarction but not to AD pathology in older persons. Neurology 67: 1960–1965. 17159101 10.1212/01.wnl.0000247053.45483.4e

[5] van Harten B, de Leeuw FE, Weinstein HC, Scheltens P, Biessels GJ (2006) Brain imaging in patients with diabetes: A systematic review. Diabetes Care 29: 2539–2548. 17065699 10.2337/dc06-1637

[6] Biessels GJ, Staekenborg S, Brunner E, Brayne C, Scheltens P (2006) Risk of dementia in diabetes mellitus: A systematic review. Lancet Neurol 5: 64–74. 16361024 10.1016/S1474-4422(05)70284-2

[7] Vagelatos NT, Eslick GD (2013) Type 2 diabetes as a risk factor for Alzheimer’s disease: the confounders, interactions, and neuropathology associated with this relationship. Epidemiol Rev 35: 152–160. 23314404 10.1093/epirev/mxs012

[8] Cukierman T, Gerstein HC, Williamson JD (2005) Cognitive decline and dementia in diabetes–systematic overview of prospective observational studies. Diabetologia 48: 2460–2469. 16283246 10.1007/s00125-005-0023-4

[9] Centers for Disease Control and Prevention (CDC), National Center for Health Statistics, Division of Health Interview Statistics, data from the National Health Interview Survey (2010) Distribution of age at diagnosis of diabetes among adult incident cases aged 18–79 years. Available: http://www.cdc.gov/diabetes/statistics/age/fig1.htm. Accessed 4 December 2012.

[10] Pavlik VN, Hyman DJ, Doody R (2005) Cardiovascular risk factors and cognitive function in adults 30–59 years of age (NHANES III). Neuroepidemiology 24: 42–50. 15459509 10.1159/000081049

[11] van Boxtel MP, Buntinx F, Houx PJ, Metsemakers JF, Knottnerus A, et al (1998) The relation between morbidity and cognitive performance in a normal aging population. J Gerontol A Biol Sci Med Sci 53: M147–54. 9520922 10.1093/gerona/53a.2.m147

[12] Spauwen PJ, Kohler S, Verhey FR, Stehouwer CD, van Boxtel MP (2012) Effects of type 2 diabetes on 12-year cognitive change: Results from the Maastricht Aging Study. Diabetes Care, Publish ahead of print, published online 28 December 2012. 10.2337/dc12-0746 PMC3661848 23275366

[13] Knopman D, Boland LL, Mosley T, Howard G, Liao D, et al (2001) Cardiovascular risk factors and cognitive decline in middle-aged adults. Neurology 56: 42–48. 11148234 10.1212/wnl.56.1.42

[14] Nooyens AC, Baan CA, Spijkerman AM, Verschuren WM (2010) Type 2 diabetes and cognitive decline in middle-aged men and women: The Doetinchem Cohort Study. Diabetes Care 33: 1964–1969. 20519662 10.2337/dc09-2038 PMC2928345

[15] Haan MN, Shemanski L, Jagust WJ, Manolio TA, Kuller L (1999) The role of APOE epsilon4 in modulating effects of other risk factors for cognitive decline in elderly persons. JAMA 282: 40–46. 10404910 10.1001/jama.282.1.40

[16] Caselli RJ, Dueck AC, Locke DE, Sabbagh MN, Ahern GL, et al (2011) Cerebrovascular risk factors and preclinical memory decline in healthy APOE epsilon4 homozygotes. Neurology 76: 1078–1084. 21325652 10.1212/WNL.0b013e318211c3ae PMC3068011

[17] Mahmoodi BK, Gansevoort RT, Veeger NJ, Matthews AG, Navis G, et al (2009) Microalbuminuria and risk of venous thromboembolism. JAMA 301: 1790–1797. 19417196 10.1001/jama.2009.565

[18] Lambers Heerspink HJ, Brantsma AH, de Zeeuw D, Bakker SJ, de Jong PE, et al (2008) Albuminuria assessed from first-morning-void urine samples versus 24-hour urine collections as a predictor of cardiovascular morbidity and mortality. Am J Epidemiol 168: 897–905. 18775924 10.1093/aje/kwn209

[19] Ruff R (1996) Ruff Figural Fluency Test: Professional manual. Lutz, FL: Psychological Assessment Resources, Inc.

[20] Ruff R, Light R, Evans R (1987) The Ruff Figural Fluency Test: A normative study with adults. Dev Neuropsychol 3: 37–51.

[21] Izaks GJ, Joosten H, Koerts J, Gansevoort RT, Slaets JP (2011) Reference data for the Ruff Figural Fluency Test stratified by age and educational level. PLoS One 6: e17045. 21347325 10.1371/journal.pone.0017045 PMC3037396

[22] Lindeboom J, Schmand B (2003) Visual Association Test. Manual. PITS B.V. Leiden, The Netherlands.

[23] Expert Committee on the Diagnosis and Classification of Diabetes Mellitus (2003) Report of the expert committee on the diagnosis and classification of diabetes mellitus. Diabetes Care 26 Suppl 1S5–20. 12502614 10.2337/diacare.26.2007.s5

[24] Monster TB, Janssen WM, de Jong PE, de Jong-van den Berg LT (2002) PREVEND Study Group Prevention of REnal and Vascular ENT Stage Disease (2002) Pharmacy data in epidemiological studies: An easy to obtain and reliable tool. Pharmacoepidemiol Drug Saf 11: 379–384. 12271879 10.1002/pds.722

[25] Izaks GJ, Gansevoort RT, van der Knaap AM, Navis G, Dullaart RP, et al (2011) The association of APOE genotype with cognitive function in persons aged 35 years or older. PLoS One 6: e27415. 22110642 10.1371/journal.pone.0027415 PMC3215744

[26] United Nations Educational, Scientific and Cultural Organization (2006) International standard classification of education ISCED 1997, May 2006 Re-edition 2006. Available: http://www.uis.unesco.org/ev.php?ID=3813_201&D2 = IDO_TOPIC. Accessed 4 December 2012.

[27] De Jong PE, Hillege HL, Pinto-Sietsma SJ, de Zeeuw D (2003) Screening for microalbuminuria in the general population: a tool to detect subjects a risk for progressive renal failure in an early phase?. Nephrol Dial Transplant 18: 10–13. 12480951 10.1093/ndt/18.1.10

[28] Bertram L, McQueen MB, Mullin K, Blacker D, Tanzi RE (2007) Systematic meta-analyses of Alzheimer disease genetic association studies: The AlzGene database. Nat Genet 39: 17–23. 17192785 10.1038/ng1934

[29] Baan CA, Nusselder WJ, Barendregt JJ, Ruwaard D, Bonneux L, et al (1999) The burden of mortality of diabetes mellitus in the Netherlands. Epidemiology 10: 184–187. 10069257

[30] Harris MI, Klein R, Welborn TA, Knuiman MW (1992) Onset of NIDDM occurs at least 4–7 yr before clinical diagnosis. Diabetes Care 15: 815–819. 1516497 10.2337/diacare.15.7.815

[31] Roger VL, Go AS, Lloyd-Jones DM, Benjamin EJ, Berry JD, et al (2012) Heart disease and stroke statistics–2012 update: A report from the American Heart Association. Circulation 125: e2–e220. 22179539 10.1161/CIR.0b013e31823ac046 PMC4440543

[32] Moreira PI, Duarte AI, Santos MS, Rego AC, Oliveira CR (2009) An integrative view of the role of oxidative stress, mitochondria and insulin in Alzheimer’s disease. J Alzheimers Dis 16: 741–761. 19387110 10.3233/JAD-2009-0972

[33] Lewington S, Whitlock G, Clarke R, Sherliker P, Emberson J, et al (2007) Blood cholesterol and vascular mortality by age, sex, and blood pressure: a meta-analysis of individual data from 61 prospective studies with 55,000 vascular deaths. Lancet 370: 1829–1839. 18061058 10.1016/S0140-6736(07)61778-4

[34] Sabayan B, Gussekloo J, de Ruijter W, Westerdorp RG, de Craen AJ (2013) Framingham stroke risk score and cognitive impairment for predicting first-time stroke in the oldest old. Stroke 44: 1866–1871. 23686982 10.1161/STROKEAHA.113.001460

[35] Bild DE, Detrano R, Peterson D, Guerci A, Liu K, et al (2005) Ethnic differences in coronary calcification: The Multi-Ethnic Study of Atherosclerosis (MESA). Circulation 111: 1313–1320. 15769774 10.1161/01.CIR.0000157730.94423.4B

[36] Sacco RL, Roberts JK, Boden-Albala B, Gu Q, Lin IF, et al (1997) Race-ethnicity and determinants of carotid atherosclerosis in a multiethnic population. the Northern Manhattan Stroke Study. Stroke 28: 929–935. 9158627 10.1161/01.str.28.5.929

[37] Launer LJ, Masaki K, Petrovitch H, Foley D, Havlik RJ (1995) The association between midlife blood pressure levels and late-life cognitive function. the Honolulu-Asia Aging Study. JAMA 274: 1846–1851. 7500533

[38] Ott A, Andersen K, Dewey ME, Letenneur L, Brayne C, et al (2004) Effect of smoking on global cognitive function in nondemented elderly. Neurology 62: 920–924. 15037693 10.1212/01.wnl.0000115110.35610.80

[39] Manolio TA, Olson J, Longstreth WT (2003) Hypertension and cognitive function: Pathophysiologic effects of hypertension on the brain. Curr Hypertens Rep 5: 255–261. 12724059 10.1007/s11906-003-0029-6

[40] Folsom AR, Rasmussen ML, Chambless LE, Howard G, Cooper LS, et al (1999) Prospective associations of fasting insulin, body fat distribution, and diabetes with risk of ischemic stroke. the Atherosclerosis Risk In Communities (ARIC) study investigators. Diabetes Care 22: 1077–1083. 10388971 10.2337/diacare.22.7.1077

[41] Craft S, Watson GS (2004) Insulin and neurodegenerative disease: Shared and specific mechanisms. Lancet Neurol 3: 169–178. 14980532 10.1016/S1474-4422(04)00681-7

[42] Cummings JL, Cole G (2002) Alzheimer disease. JAMA 287: 2335–2338. 11988038 10.1001/jama.287.18.2335

[43] Gispen WH, Biessels GJ (2000) Cognition and synaptic plasticity in diabetes mellitus. Trends Neurosci 23: 542–549. 11074263 10.1016/s0166-2236(00)01656-8

[44] Bu G (2009) Apolipoprotein E and its receptors in alzheimer's disease: Pathways, pathogenesis and therapy. Nat Rev Neurosci 10: 333–344. 19339974 10.1038/nrn2620 PMC2908393

[45] Matthews FE, Chatfield M, Brayn C, Medical Research Council Cognitive Function, Ageing Study (2006) An investigation of whether factors associated with short-term attrition change or persist over ten years: data from the Medical Research Council Cognitive Function and Ageing Study (MRC CFAS). BMC Public Health 6: 185. 16848886 10.1186/1471-2458-6-185 PMC1538586

[46] Joosten H, Izaks GJ, Slaets JP, de Jong PE, Visser ST, et al (2011) Association of cognitive function with albuminuria and eGFR in the general population. Clin J Am Soc Nephrol 6: 1400–1409. 21566108 10.2215/CJN.05530610 PMC3109938

